# Comparative Evaluation of Standard Cholangiography, Intravenous, and Intracholecystic Indocyanine Green Fluorescence Cholangiography During Elective Laparoscopic Cholecystectomy: Results of a Three-Arm Randomized Trial

**DOI:** 10.3390/medicina62030515

**Published:** 2026-03-10

**Authors:** Savvas Symeonidis, Ioannis Mantzoros, Orestis Ioannidis, Elissavet Anestiadou, Angeliki Koltsida, Panagiotis Christidis, Stefanos Bitsianis, Trigona Karastergiou, Stylianos Apostolidis, Vasileios Foutsitzis, Efstathios Kotidis, Manousos-Georgios Pramateftakis, Stamatios Angelopoulos

**Affiliations:** 1 4th Department of General Surgery, General Hospital of Thessaloniki “G. Papanikolaou”, Aristotle University of Thessaloniki, 54124 Thessaloniki, Greece; simeonidissavvas@yahoo.com (S.S.); imanvol@gmail.com (I.M.); elissavetxatz@gmail.com (E.A.); aggeliki.koltsida@gmail.com (A.K.); panagiotischristidis13@gmail.com (P.C.); sbitsiani@gmail.com (S.B.); n.karastergiou@gmail.com (T.K.); vafoutsitzis@yahoo.com (V.F.); skotidis@gmail.com (E.K.); mpramateftakis@hotmail.com (M.-G.P.); saggelopoulos@auth.gr (S.A.); 2 1st Propaedeutic Department of Surgery, AHEPA University Hospital, Aristotle University of Thessaloniki, 54636 Thessaloniki, Greece; stlsa@auth.gr

**Keywords:** laparoscopic cholecystectomy, intraoperative cholangiography, indocyanine green, fluorescence cholangiography, intracholecystic ICG, biliary anatomy, randomized controlled trial, surgeon satisfaction

## Abstract

*Background and Objectives*: Bile duct injury is a relatively rare, but critical complication of laparoscopic cholecystectomy and is most commonly attributed to misinterpretation of biliary anatomy. Intraoperative biliary imaging may enhance anatomical recognition and reduce operative uncertainty, yet the optimal imaging modality remains debated. This study aimed to compare conventional intraoperative X-ray cholangiography with two fluorescence-based techniques—intravenous and intracholecystic indocyanine green fluorescence cholangiography—with respect to biliary visualization, perioperative outcomes, and surgeon satisfaction during elective laparoscopic cholecystectomy. *Materials and Methods*: This prospective, single-center, single-blind randomized controlled trial included 240 adult patients scheduled for elective laparoscopic cholecystectomy between June 2021 and December 2022. Participants were randomized equally to standard intraoperative cholangiography, intravenous indocyanine green fluorescence cholangiography, or intracholecystic indocyanine green fluorescence cholangiography. The primary outcome was successful visualization of predefined extrahepatic biliary landmarks, including the critical junction. Secondary outcomes included cholangiography duration, perioperative complications, postoperative inflammatory markers, and surgeon satisfaction assessed using a five-point Likert scale. This study was registered at ClinicalTrials.gov (NCT04908826). *Results*: Visualization rates of the critical junction and major extrahepatic bile ducts were comparable among three groups, with no statistically significant differences observed. Both fluorescence-based techniques achieved a 100% technical success rate, whereas standard cholangiography failed in a small proportion of cases. Cholangiography duration was significantly shorter in the fluorescence groups compared with standard cholangiography (*p* < 0.001). Surgeon satisfaction scores were significantly higher for both fluorescence approaches, with a slight preference for intravenous administration. Perioperative complication rates and postoperative inflammatory markers were com-parable among groups. *Conclusions*: Intravenous and intracholecystic indocyanine green fluorescence cholangiography are non-inferior to conventional intraoperative cholangiography for biliary anatomy visualization and offer advantages in procedural efficiency and surgeon satisfaction. Fluorescence-based imaging represents a safe and effective alternative for intraoperative biliary mapping during elective laparoscopic cholecystectomy.

## 1. Introduction

Gallstones or cholelithiasis is a chronic, relapsing hepatobiliary disorder arising from dysregulation of cholesterol, bilirubin, and bile acid metabolism, which promotes gallstone formation within the intrahepatic bile ducts, extrahepatic biliary tree, or gallbladder and represents one of the most prevalent gastrointestinal diseases worldwide, affecting 10–20% of the adult population in Western countries [1]. Acute cholecystitis, the most common complication of cholelithiasis, constitutes a leading cause of hospital admission in Europe, and its management has evolved through several surgical approaches over time. Apart from acute cholecystitis, a major concern in gallstone disease is the migration of stones from the gallbladder into the extrahepatic biliary tree. This process—commonly resulting in choledocholithiasis—is observed in a significant subset of patients with gallstones and may lead to serious downstream complications, including obstructive jaundice, acute ascending cholangitis, and gallstone pancreatitis. These conditions can vary in severity from edematous inflammation to necrotizing or hemorrhagic pancreatitis, the latter of which carries substantial morbidity and mortality and may be life-threatening if not promptly recognized and treated [2]. Notably, certain stone morphologies—such as small, spiculated pigment stones—are more prone to migrate and obstruct the common bile duct, thereby increasing the risk of such complications. This phenomenon has been highlighted in recent studies linking stone composition and shape to a higher likelihood of downstream biliary obstruction and severe clinical sequelae [3]. Laparoscopic cholecystectomy (LC) is currently considered the gold-standard treatment strategy for symptomatic gallbladder disease and more than 900,000 cholecystectomies are conducted each year in Europe [4]. The widespread adoption of the laparoscopic approach is supported by low mortality and morbidity rates, reported at approximately 0.08–0.14% and 1.6–5.3%, respectively, while conversion to open surgery is necessary in 4–6% of cases [5]. Although LC is widely regarded as a safe procedure, entails a wide spectrum of technical challenges. Its adoption has been accompanied by a significant increase in the incidence of bile duct injuries (BDI), with reported rates ranging from 0.3% to 1.8%, markedly exceeding the 0.1–0.2% historically observed in open cholecystectomy [5,6]. These injuries are associated with substantial clinical burden, including prolonged hospital stay, need for complex biliary reconstruction, recurrent episodes of cholangitis or biliary strictures, and a significant negative impact on long-term health-related quality of life, with mortality reported in the most severe cases [7]. Initially, five mechanisms of BDI were described in open cholecystectomy: inadvertent division of the incorrect duct, occlusion of the common bile duct during cystic duct ligation, ischemic damage from excessive dissection, mechanical injury during ductal exploration or forced dilatation, and thermal injury related to inappropriate energy use [8]. Although this classification provides a useful framework, bile duct injuries during laparoscopic cholecystectomy can be more pragmatically grouped into two fundamental categories: errors arising from misidentification of biliary anatomy, in up to 71–97% of cases, and those resulting from technical or operative mishaps [9]. Additional contributors for a difficult LC predisposing to BDI include acute or chronic inflammation, obesity, aberrant biliary anatomy, limited surgeon experience, time pressure, and adverse intraoperative conditions, all of which increase cognitive load and the likelihood of error [10].

Given the profound implications of these complications, prevention of BDI remains a central priority in hepatobiliary surgery. The principle of the “critical view of safety” (CVS), first described by Strasberg and colleagues, is universally regarded as an essential step in the safe performance of LC that has been shown to significantly reduce the risk of ductal misidentification when rigorously achieved and documented [11]. However, CVS is not infallible, particularly in the presence of severe inflammation or distorted anatomy, and may not be achievable in a substantial proportion of difficult cases. Consequently, data regarding the adjunctive use of intraoperative biliary imaging modalities, including routine or selective intraoperative cholangiography, fluorescent indocyanine green cholangiography, and near-infrared imaging is gaining territory in the worldwide literature, to enhance real-time anatomical visualization and further mitigate the risk of bile duct injury [12].

Intraoperative cholangiography (IOC) has traditionally been employed to visualize the extrahepatic biliary anatomy, detect choledocholithiasis, and identify anatomical variants or pathological conditions such as biliary fistulas [13]. The routine performance of IOC during LC has been associated with a low incidence of bile duct injury and enabling prompt intraoperative detection and repair, resulting in favorable outcomes [14]. Despite its established utility, IOC is associated with several drawbacks that may increase operative complexity and physiological stress, including prolonged operative time, the need for specialized personnel and portable radiographic equipment, exposure to ionizing radiation, cystic duct cannulation, and increased procedural costs. Moreover, IOC does not unequivocally prevent bile duct injury and, in certain cases, may itself contribute to ductal trauma, particularly during cannulation of the cystic duct [15,16].

Indocyanine green (ICG) is a water soluble, fluorescent tricarbocyanine compound that is excited by near-infrared light, with peak absorption and emission in the range of approximately 805–835 nm. After intravenous administration, it binds rapidly to plasma proteins, is metabolized in the liver and excreted almost exclusively via bile, allowing for intraoperative visualization of biliary anatomy when coupled with dedicated fluorescence imaging platforms [15]. ICG enables real-time identification of key anatomical structures such as the biliary tract, ureters, parathyroid glands, and thoracic duct while also allowing for intraoperative assessment of tissue perfusion in a wide range of procedures including colorectal, esophageal, gastric, bariatric, hepatobiliary, reconstructive, and emergency surgery for ischemic or strangulated conditions. In addition, it supports tumor localization in organs such as the liver, pancreas, adrenal glands, and retroperitoneum, facilitates the detection of peritoneal implants, and plays an important role in sentinel lymph node identification and lymphatic mapping in malignancies of the gastrointestinal tract, breast, esophagus, and skin [17]. In 2009, Ishizawa and colleagues were the first to repot ICG administration for intraoperative biliary mapping in a series of patients including hepatectomies and open cholecystectomies, and subsequent clinical experience has confirmed its excellent safety profile, with iodine hypersensitivity representing the principal contraindication [18,19]. A recent meta-analysis of eight randomized controlled trials including 1586 patients demonstrated that indocyanine green fluorescence cholangiography (ICG-FC) significantly increased the success rate of common bile duct identification during LC, although no significant differences were observed for bile duct injury rates or visualization of other biliary structures. Trial sequential analysis confirmed that the improvement in common bile duct identification was unlikely to represent a type I error, but further trials are required to clarify its impact on additional outcomes [20]. On the contrary, in the VIFCAL randomized controlled trial including 134 patients with grade I–II acute lithiasic cholecystitis, intravenous indocyanine green fluorescence cholangiography did not reduce operative time to achieve the CVS nor improve overall visualization of biliary structures compared with conventional intraoperative cholangiography, and no bile duct injuries occurred in either group [21]. The authors concluded that, in the setting of acute inflammation, ICG provided suboptimal biliary contrast and could not reliably replace IOC for safe anatomical identification.

More recently, direct administration of ICG into the gallbladder was described in animal experimental models [22] and was first reported in humans in 2017 [23]. Although this modification of near-infrared cholangiography (NIRC) technique has recently been introduced as a technical modification, and the current body of evidence supporting its use remains limited [24,25,26]. Preliminary data suggest potential advantages over intravenous ICG administration in selected cases, enabling direct visualization of the biliary anatomy while minimizing interference from background hepatic fluorescence [24]. However, additional well-designed studies are to be performed to better define the role and the optimal route of ICG administration in acute and complex clinical settings and to establish the patient and procedural factors that maximize its diagnostic performance.

Building on our previous randomized evaluation of intraoperative biliary mapping techniques, which demonstrated that indocyanine green fluorescence cholangiography provides biliary visualization comparable to conventional intraoperative cholangiography while improving surgeon satisfaction [27], and in order to address the aforementioned need, the present study was designed to further refine and expand these observations by exploring alternative routes of indocyanine green administration and their impact on perioperative performance.

In order to address this need, the present single-center randomized controlled trial was designed to compare the efficacy and peri- and postoperative outcomes of two methods of indocyanine green administration—intravenous and intragallbladder—both against each other and in comparison with conventional X-ray intraoperative cholangiography (IOC). The primary aims were to assess the quality of extrahepatic biliary anatomy visualization, perioperative results, and surgeon satisfaction during elective laparoscopic cholecystectomy.

## 2. Materials and Methods

### 2.1. Study Design

This prospective, single-center, single-blind randomized controlled trial enrolled 240 patients scheduled for elective LC, who were evenly randomized to three groups of 80 participants each. Patients in Group A underwent conventional intraoperative X-ray cholangiography (IOC), those in Group B received intravenous indocyanine green fluorescence cholangiography after preoperative administration of 0.3 mg/kg ICG six hours prior to surgery, while in Group C, fluorescent cholangiography was performed following direct intracholecystic injection of ICG at a dose of 0.03 mg/kg.

All participants were managed at a tertiary academic referral center in Greece between June 2021 and December 2022. Indications for surgery included cholelithiasis or uncomplicated gallstone disease. Patients were eligible if they were older than 18 years, had provided written informed consent following thorough preoperative counseling, and were scheduled for elective LC. Exclusion criteria comprised referred iodine hypersensitivity, pregnancy or lactation, thyroid disorders, renal or hepatic impairment, suspected gallbladder malignancy, urgent or emergency presentation, or unwillingness to participate in this study. Cases of suspected choledocholithiasis or cholangitis was evaluated preoperatively with ultrasonography and MRCP, followed by ERCP when indicated. The protocol adhered to the Declaration of Helsinki and was registered on ClinicalTrials.gov (NCT04908826, Registration date 26 May 2021).

A pre-designed data extraction form was used to record demographic variables, operative parameters, laboratory markers, visualization rates, perioperative complications, cholangiography duration, and surgeon satisfaction prospectively. All surgical procedures were performed by three consultant surgeons with strong background in advanced hepatobiliary laparoscopy, strictly applying the critical view of safety (CVS) technique. Last but not least, all patients received antibiotic prophylaxis and were followed clinically for six months postoperatively through scheduled outpatient visits and/or telephone communication to assess for late biliary symptoms, readmissions, or reinterventions.

### 2.2. Randomization and Blinding

After enrollment, a secure electronic system was used to randomize patients to one of the three study arms. Allocation was concealed from participants, while all patient data were anonymized before analysis. Written informed consent was obtained by the investigators.

### 2.3. Surgical Approach and Imaging Protocols

All procedures followed the standard four-port American technique, with the patient placed in supine position.

Group A: X-ray intraoperative cholangiography (IOC)

After careful dissection of Calot’s triangle and unequivocal identification of the cystic duct and cystic artery, a titanium clip was placed proximally at the transition between the cystic duct and the gallbladder infundibulum. A partial circumferential incision was then made on the cystic duct distal to the clip using laparoscopic scissors to allow for selective access. A 5-Fr cholangiography catheter (Cook Medical, Bloomington, IN, USA) was gently advanced into the cystic duct with the assistance of a laparoscopic grasper. Correct catheter positioning and integrity were verified by flushing 3 mL of isotonic saline through the catheter to exclude leakage.

A mobile C-arm fluoroscopy system (Ziehm Imaging, Nürnberg, Germany) was positioned over the right upper abdominal quadrant. Contrast imaging was performed by injecting a mixture of 10 mL of iobitridol (Xenetix^®^, Guerbet, Roissy-Charles-de-Gaulle Cedex, France) diluted with 10 mL of 0.9% saline through the catheter until satisfactory opacification of the extrahepatic biliary tree was achieved. The duration of IOC was defined as the interval between catheter insertion and removal after acquisition of adequate dynamic fluoroscopic images.

Group B: Intravenous fluorescence cholangiography

Under aseptic conditions, one vial of ICG VERDYE (Diagnostic Green GmbH, Aschheim-Dornach, Germany), containing 25 mg of indocyanine green (ICG), was diluted with 10 mL of sterile water for injection to obtain a final concentration of 2.5 mg/mL, in accordance with the manufacturer’s recommendations. Six hours before induction of anesthesia, ICG was administered intravenously at a dose of 0.3 mg/kg via either a peripheral or central venous line, followed by a 10 mL saline flush.

**Figure 1:**
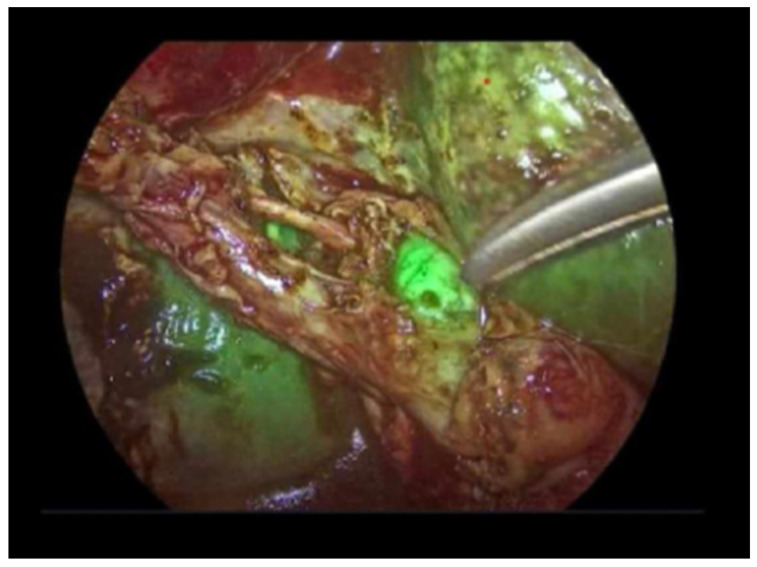
Intraoperative near-infrared fluorescence cholangiography following intravenous administration of indocyanine green. Fluorescence imaging demonstrates visualization of the biliary anatomy in the presence of intense hepatic parenchymal fluorescence, which partially obscures delineation of the extrahepatic bile ducts.

Laparoscopic procedures were performed using a KARL STORZ system (Tuttlingen, Germany) equipped with an IMAGE 1 S high-definition camera capable of both white-light and near-infrared fluorescence imaging. Following trocar placement and initial exploration of the peritoneal cavity, the operative field was assessed sequentially under standard white-light illumination and fluorescence mode prior to dissection of Calot’s triangle, enabling early identification of biliary landmarks. Fluorescence imaging was subsequently used during dissection to enhance visualization of the extrahepatic biliary tree and to confirm achievement of the Critical View of Safety. The duration of fluorescence cholangiography was defined as the cumulative time during which the near-infrared imaging mode was activated (Figure 1).

Group C: Intracholecystic fluorescence cholangiography

**Figure 2:**
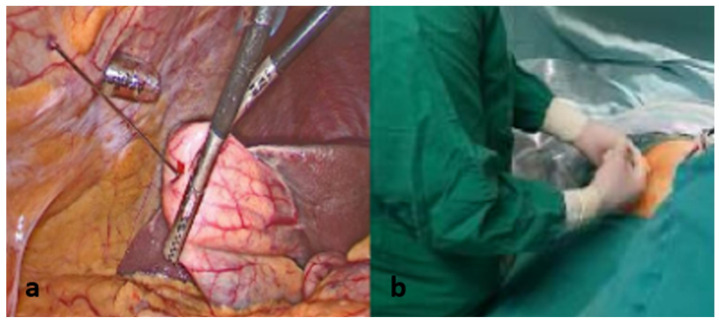
Intracholecystic indocyanine green fluorescence cholangiography. (**a**) Intraoperative view demonstrating direct injection of indocyanine green into the gallbladder following partial exposure and controlled puncture. (**b**) Preparation and administration of indocyanine green for intragallbladder injection under sterile conditions prior to fluorescence imaging.

**Figure 3:**
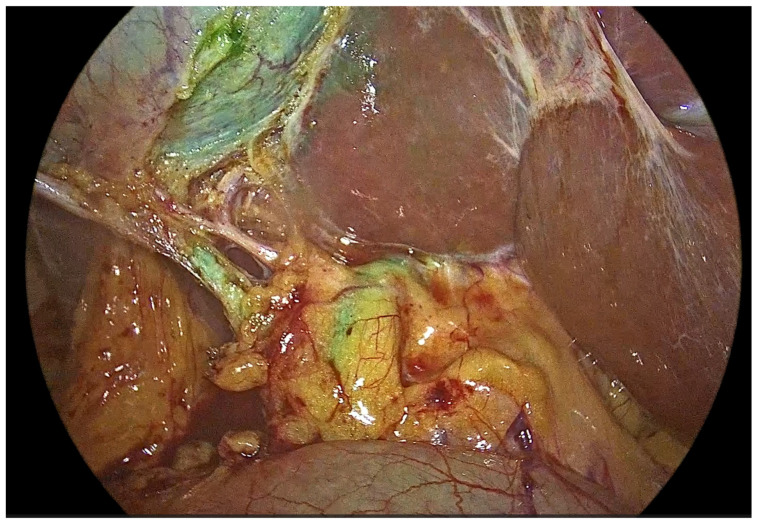
Intraoperative near-infrared fluorescence cholangiography following intragallbladder administration of indocyanine green. Fluorescence imaging demonstrates selective opacification of the biliary tree, allowing for clear delineation of the cystic duct, cystohepatic junction, and extrahepatic bile ducts without interference from background hepatic fluorescence.

With continuous laparoscopic visualization, the gallbladder fundus was punctured percutaneously using a 25-gauge epidural needle (Spinocan^®^, B. Braun Melsungen AG, Melsungen, Germany) (Figure 2). Subsequently, 2.5 mL of the prepared ICG solution was injected directly into the gallbladder lumen. After needle withdrawal, the puncture site was immediately secured using laparoscopic graspers to minimize bile leakage. Following a waiting period of approximately three minutes, near-infrared fluorescence imaging was initiated to perform intracholecystic fluorescent cholangiography. Once the biliary anatomy was adequately delineated, the Critical View of Safety was confirmed (Figure 3).

### 2.4. Statistical Analysis

Normality of data distribution was evaluated using the Shapiro–Wilk test. Continuous variables exhibiting normal distribution were presented as mean ± standard deviation, whereas non-normally distributed variables were expressed as median with interquartile range. Categorical data were summarized as absolute numbers and percentages. Statistical significance was assessed using a two-sided threshold of *p* < 0.05, with a 95% confidence interval applied throughout the analysis.

For comparisons of continuous variables among the three independent study groups, one-way analysis of variance (ANOVA) was used for normally distributed data. In cases of non-normal distribution, the Kruskal–Wallis test was applied. When statistically significant differences were identified, appropriate post hoc pairwise comparisons were performed. Within-group comparisons between pre- and postoperative measurements were analyzed using paired Student’s t-tests or the Wilcoxon signed-rank test, as appropriate. Associations between categorical variables were examined using the chi-square (χ^2^) test. All analyses were conducted using Jamovi software (version 1.6.18.0; The jamovi Project, Sydney, Australia).

### 2.5. Outcomes

The primary outcome measure was the rate of successful visualization of predefined biliary anatomical landmarks. These landmarks included the cystohepatic junction, defined as clear identification of at least 1 cm of the cystic duct, common hepatic duct, and common bile duct distal to their confluence; the common hepatic duct from the cystohepatic junction to its bifurcation; the common bile duct from the cystohepatic junction to the retroduodenal segment; and the cystic duct from the cystohepatic junction to its origin at the gallbladder. Additional primary endpoints included markers of the inflammatory response, duration of cholangiography, and surgeon satisfaction. Surgeon satisfaction was assessed immediately after surgery using a 5-point Likert scale, ranging from 1 (very poor) to 5 (excellent).

Secondary outcomes comprised intraoperative complications, complications occurring on postoperative day 1, rates of unsuccessful cholangiography, and preoperative and postoperative (day 1) renal, hepatic, and coagulation laboratory parameters.

## 3. Results

### 3.1. Study Population and Baseline Characteristics

**Figure 4:**
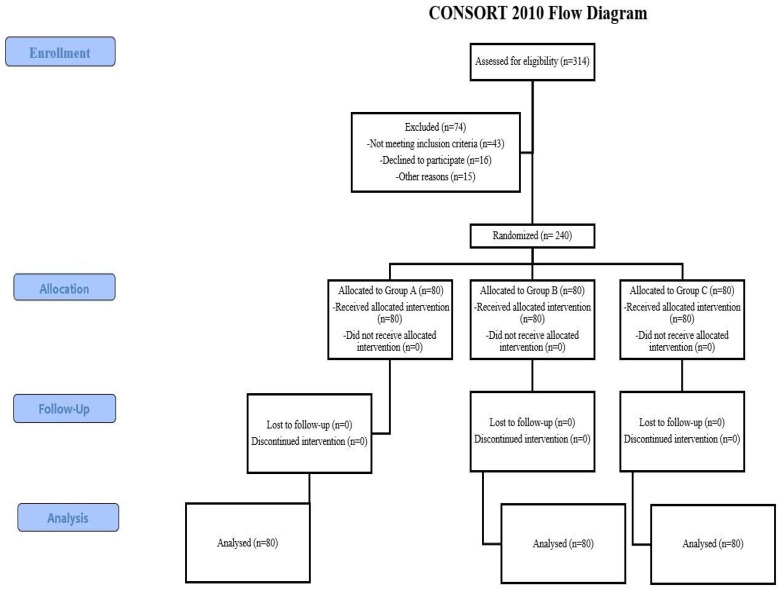
CONSORT 2010 diagram of the trial.

A total of 314 patients were evaluated for study participation, of whom 240 were ultimately included (Figure 4). Exclusions were primarily due to consent withdrawal, lack of written informed consent, or known iodine hypersensitivity. After randomization, each treatment group comprised 80 patients, all of whom were analyzed.

Baseline demographic and clinical characteristics were comparable across the three study groups. No statistically significant differences were observed in age, sex distribution, body mass index (BMI), or ASA classification (Table 1). Surgical indications were comparable among groups, with cholelithiasis representing the predominant indication. Intracholecystic fluorescence cholangiography was not applied in cases of gallbladder polyps.

Mean operative duration did not differ significantly between the three groups (*p* = 0.858), and overall perioperative complication rates were comparable. During the six-month clinical follow-up period, no patients developed symptoms suggestive of delayed bile duct injury, biliary stricture, or required reintervention.

### 3.2. Feasibility and Success of Cholangiography Techniques

Standard intraoperative cholangiography (IOC) could not be completed in 3 out of 80 patients (3.75%) due to cystic duct obstruction. These cases required conversion to open cholecystectomy. In contrast, both intravenous and intracholecystic ICG fluorescence cholangiography were successfully completed in all patients (100% technical success).

### 3.3. Visualization of Extrahepatic Biliary Anatomy

Visualization rates of key biliary structures were comparable among the three techniques. No statistically significant differences were identified in the visualization of the critical junction (*p* = 0.737), common hepatic duct (*p* = 0.543), common bile duct (*p* = 0.106), or cystic duct (*p* = 0.234). Detailed visualization rates are presented in Table 2.

### 3.4. Cholangiography Duration and Operative Time

A statistically significant difference was identified in cholangiography time, which was markedly longer in Group A compared with Group B and Group C (305 ± 18.6 s vs. 110 ± 11.1 s vs. 125 ± 13.6 s, *p* < 0.001). This discrepancy did not translate into a difference in total operative duration, encompassing both the cholecystectomy and cholangiography components. (47.1 ± 7.31 min vs. 46.5 ± 7.43 min vs. 47.3 ± 6.86 min, *p* = 0.858). Surgeon-related ease and feasibility of intraoperative cholangiography, assessed using a 1–5 numerical scale, demonstrated a significant advantage for intravenous ICG fluorescence cholangiography and intracholecystic approach with a slight preference for the intravenous one (2.9 ± 0.79 vs. 4.1 ± 1.05 vs. 4 ± 0.91 for groups A, B and C, respectively; *p* < 0.001).

### 3.5. Surgeon Satisfaction

Surgeon-reported satisfaction scores differed significantly between techniques (*p* < 0.001). Both fluorescence-based approaches were rated higher than standard IOC, with intravenous ICG-FC receiving the highest satisfaction scores, followed closely by the intracholecystic approach (Table 2).

### 3.6. Postoperative Inflammatory Response

Intergroup comparisons of postoperative inflammatory markers are summarized in Table 3. A statistically significant difference was observed only for postoperative white blood cell (WBC) count, which was higher in the IOC group compared with both fluorescence groups (*p* = 0.045). No significant differences were detected in CRP, IL-6, or TNF-α levels.

### 3.7. Postoperative Hepatic, Renal, and Coagulation Parameters

Postoperative hepatic and renal function tests on postoperative day one were largely comparable among groups. Direct bilirubin levels were significantly higher in the IOC group (*p* = 0.001), whereas no differences were observed for transaminases, alkaline phosphatase, γ-GT, total bilirubin, coagulation parameters, or renal function indices.

### 3.8. Postoperative Complications and Detection of Choledocholithiasis

Thirty-day postoperative complication rates did not differ significantly among the three groups (*p* = 0.305). No bile duct injuries were recorded. All complications were classified as Clavien–Dindo grade II and were managed conservatively.

Intraoperative choledocholithiasis was identified in five patients overall (two in Group A, one in Group B, and two in Group C). All patients underwent postoperative ERCP with successful stone extraction and uneventful recovery.

## 4. Discussion

First performed in 1985 by Professor Mühe in Germany, laparoscopic cholecystectomy (LC) quickly emerged as a transformative procedure and gained worldwide recognition [28]. Nowadays, LC represents the cornerstone of surgical management for benign gallbladder disease and is among the most frequently performed operations in general surgery worldwide, both for emergent and elective cases [29]. However, despite its established benefits and widespread implementation, the shift from open to laparoscopic techniques has been accompanied by an unintended increase in both the incidence and severity of bile duct injuries (BDIs). Contemporary series report BDI rates of up to 0.6% following laparoscopic cholecystectomy, compared with approximately 0.1% in the era of open surgery [30]. Such injuries may result in serious clinical sequelae, including bile leakage, biliary peritonitis, cholangitis, and the development of secondary biliary cirrhosis, while long-term outcomes often involve recurrent biliary strictures and the requirement for complex reconstructive procedures [31]. BDIs are associated with significant clinical morbidity, prolonged hospitalization, need for complex reconstructive procedures, and substantial medicolegal and socioeconomic consequences. This burden is further amplified by the high prevalence of gallstone disease, which affects tens of millions of individuals globally, including nearly 20 million in the United States alone [32].

Most BDIs are identified intraoperatively or shortly after surgery, most often presenting as bile leakage or biliary obstruction. However, a proportion of injuries become evident later in the postoperative course, which may result in delayed or inappropriate management, particularly when patients require referral from secondary hospitals to tertiary centers for definitive treatment [33]. BDIs arise from a combination of anatomical, surgeon-related, and patient-related factors, with anatomical misidentification representing the predominant cause. Failure to correctly recognize biliary anatomy, widely referred to as misidentification injury, accounts for approximately 71–79% of reported injuries and is most commonly related to anatomical variations and errors in cognitive perception rather than from inadequate technical skill [10]. This mechanism usually includes erroneous recognition of the common bile duct or an aberrant right hepatic duct as the cystic duct. Such errors are often facilitated by distorted anatomy secondary to inflammation, fibrosis, anatomical variation, or excessive traction on the gallbladder. However, it is important to recognize that bile duct injuries and other perioperative complications were not exclusive to the laparoscopic era. Open cholecystectomy was likewise associated with a spectrum of adverse events, including biliary injury, hemorrhage, wound infections, incisional hernias, and prolonged postoperative recovery [34]. Although the initial transition to laparoscopy was accompanied by a transient increase in bile duct injury rates, the minimally invasive approach has ultimately proven superior in terms of reduced postoperative pain, shorter hospital stay, faster return to normal activity, lower wound-related morbidity, and improved overall quality of life [35]. Consequently, laparoscopic cholecystectomy represents a major advancement in the management of gallstone disease, offering clear short- and long-term benefits compared with the traditional open technique.

Recognizing the above BDI mechanisms has shifted the focus of BDI prevention from purely technical refinements to strategies aimed at improving anatomical cognition and situational awareness [36]. Consequently, international guidelines strongly advocate for systematic identification of biliary anatomy and strict adherence to the critical view of safety as the cornerstone of safe cholecystectomy [37]. Beyond these foundational principles, several adjunctive intraoperative imaging techniques—including intraoperative cholangiography, laparoscopic ultrasonography, and near-infrared indocyanine green (NIR-ICG) fluorescence cholangiography—have been developed to provide real-time anatomical confirmation, enhance spatial orientation, and offer an additional safeguard against misinterpretation, particularly in complex or anatomically challenging cases [38].

Traditionally, intraoperative cholangiography (IOC) has been employed during cholecystectomy to identify common bile duct stones and delineate biliary anatomy, reflecting a perception of improved surgical safety [39]. The procedure involves dissection of Calot’s triangle, cannulation of the cystic duct, and contrast injection to obtain real-time fluoroscopic imaging. IOC offers several clinical advantages, including early recognition of bile duct injuries and detection of choledocholithiasis; however, its routine use remains limited, with most surgeons favoring a selective application. However, its use has progressively declined and varies widely among surgeons, ranging from routine application to complete omission [40]. This variability is likely related to the additional operative time required, technical difficulty—particularly in acute cholecystitis—and the lack of uniform management pathways for detected ductal stones. Moreover, the clinical value of IOC has increasingly been questioned in the context of advances in preoperative imaging, including magnetic resonance cholangiopancreatography, as well as the broad availability of endoscopic ultrasound, endoscopic retrograde cholangiopancreatography, and fluorescence-based cholangiography techniques [41].

Indocyanine green fluorescence cholangiography (ICG-FC) has gained attention as a reliable method for real-time intraoperative identification of vasculo-biliary anatomy, demonstrating high visualization rates of the cystic duct, common bile duct, cystic artery and aberrations [42]. Indocyanine green is a sterile, water-soluble anionic dye with a molecular weight of 776 Da that, following intravenous administration, rapidly binds to plasma proteins and is taken up by the liver without metabolic alteration, being almost exclusively excreted into bile. Under near-infrared illumination, ICG exhibits characteristic fluorescence, enabling visualization of vascular and biliary structures using dedicated imaging systems [43].Unlike intraoperative cholangiography, ICG-FC avoids cystic duct cannulation and radiation exposure and can be seamlessly integrated into the surgical workflow without prolonging operative time. Continuous fluorescence imaging enhances spatial orientation and may reduce the risk of bile duct injury while also serving as a valuable adjunct in surgical training [44]. Although improved resident performance has been reported with ICG-FC, all procedures in the present study were performed by experienced consultant surgeons to limit operator-dependent variability [45].

Currently, there is no universally accepted protocol regarding the optimal dose or timing of ICG administration, although most published studies advocate administration of 2.5 mg administered 2–6 h preoperatively [46]. However, despite its widespread adoption, ICG-FC presents several inherent limitations that may compromise its effectiveness in certain clinical scenarios. Following systemic administration, ICG rapidly accumulates within the hepatic parenchyma, generating a high background fluorescence signal that can obscure biliary structures and reduce the bile duct–to–liver contrast, particularly when imaging is performed shortly after injection [47]. This effect necessitates careful preoperative timing, which varies among patients and limits the utility of intravenous ICG in emergency or unplanned cholecystectomies. Furthermore, fluorescence signal penetration is restricted to a depth of approximately 5–10 mm, reducing visualization accuracy in patients with obesity, abundant fatty tissue, or dense inflammatory and fibrotic changes [48]. Although liver fluorescence was not quantitatively assessed in this study, surgeon satisfaction scores suggest that it did not significantly interfere with biliary visualization. Additional factors, including hepatic dysfunction, impaired biliary excretion, obesity, and fibrotic tissue associated with recurrent inflammation, may further limit fluorescence signal penetration, which is generally restricted to depths approximately 10 mm [49].

To address these limitations, direct intracholecystic ICG administration has been proposed as an alternative approach to near-infrared fluorescent cholangiography, aiming to overcome the limitations of intravenous ICG delivery [23]. By injecting ICG directly into the gallbladder lumen, fluorescence is confined primarily to the biliary tree, resulting in a markedly improved bile duct–to–liver contrast and minimizing background hepatic fluorescence. Available clinical evidence, although limited, suggests that this technique enables reliable visualization of key biliary structures—including the cystic duct, common bile duct, and their confluence—particularly before dissection and in the presence of inflammation or fibrotic distortion [50]. Moreover, intragallbladder ICG injection allows for immediate real-time imaging without the need for preoperative timing or higher systemic doses, making it especially attractive in acute or unplanned settings [51]. While reported outcomes indicate favorable safety and feasibility, concerns remain regarding dye spillage, technical variability, and lack of standardization in dosing and administration technique, underscoring the need for further prospective studies and consensus guidelines before widespread adoption.

To the best of our knowledge, this is the first prospective study to directly compare three intraoperative biliary imaging modalities—standard intraoperative cholangiography (IOC), intravenous NIR-ICG fluorescence cholangiography, and intracholecystic NIR-ICG fluorescence cholangiography—within a single study design. This head-to-head comparison allowed for a standardized evaluation of biliary visualization efficacy and procedural performance across techniques under identical operative conditions, providing novel comparative data that are currently lacking in the literature. Our study demonstrated that both intravenous and intracholecystic near-infrared indocyanine green fluorescence cholangiography were non-inferior to IOC in visualizing key components of the extrahepatic biliary tree. Specifically, no statistically significant differences were observed among the three groups in identifying the cystic duct–common bile duct junction, cystic duct, common bile duct, or common hepatic duct, consistent with findings reported in prior systematic reviews. Although some studies have suggested superior visualization rates with ICG-FC, particularly for the common hepatic duct, these differences have not consistently reached statistical significance [52].

Postoperative assessment for biliary injury or obstruction routinely relies on biochemical evaluation of hepatic function [53]. In the present study, postoperative liver enzymes and bilirubin levels were largely comparable across the three groups, with the exception of higher direct bilirubin values observed on the first postoperative day in the IOC cohort. This isolated finding did not translate into clinically relevant hepatic dysfunction and suggests that fluorescence-based cholangiography does not impose an additional risk of biliary obstruction or hepatocellular injury. Transient alterations in liver function following laparoscopic cholecystectomy have previously been attributed to pneumoperitoneum, surgical manipulation, and hemodynamic changes affecting hepatic perfusion [54]. Consistent with prior reports, including the study by Hasukić et al. [55], no significant intergroup differences were identified in coagulation parameters in our cohort, indicating preserved hepatic synthetic function. Furthermore, postoperative renal function remained stable across all groups, supporting the renal safety of both intraoperative cholangiography and fluorescence-based imaging techniques.

Laparoscopic cholecystectomy nonetheless elicits an acute-phase endocrine and immune response, reflecting a transient state of systemic inflammation that may be accompanied by cytokine-mediated anti-anabolic or catabolic effects, even within the context of minimally invasive surgery [56]. Compared with open cholecystectomy, the laparoscopic approach is associated with reduced surgical stress and a blunted inflammatory response, translating into lower postoperative morbidity, shorter hospital stays, and reduced healthcare costs; modulation of the pro-inflammatory cascade has also been linked to reduced postoperative pain. To our knowledge, the impact of bile duct injury prevention and detection techniques—such as intraoperative cholangiography and near-infrared indocyanine green fluorescence cholangiography—on postoperative inflammatory responses has not previously been evaluated. In the present study, although white blood cell counts on the first postoperative day were higher in the IOC group, values remained within physiological limits, and no clinically meaningful inflammatory differences were observed. Collectively, these findings indicate that neither biliary mapping modality confers a significant additional inflammatory burden.

Although patient-reported outcomes and satisfaction following laparoscopic cholecystectomy have been extensively investigated, surgeon-reported satisfaction and perceived usability of intraoperative biliary mapping techniques remain relatively underreported in the literature [57,58]. Intraoperative biliary mapping techniques aim to enhance anatomical orientation and may contribute to reduced complication rates by facilitating accurate identification of critical structures [59]. Previous studies have assessed the usability and image quality of different cholangiography modalities using a variety of evaluation scales. In a blinded non-inferiority randomized controlled trial including 120 patients undergoing elective laparoscopic cholecystectomy, fluorescence cholangiography using indocyanine green was non-inferior to X-ray cholangiography in visualizing the critical junction between the cystic, common hepatic, and common bile ducts [60]. Notably, fluorescence cholangiography required significantly less operative time, supporting its role as an efficient and non-invasive alternative for intraoperative biliary imaging [60]. Based on a literature review by Symeonidis et al. [61], intragallbladder indocyanine green (ICG) fluorescence cholangiography appears to be associated with high intraoperative acceptance, largely due to its immediate and intuitive visualization of biliary anatomy. The technique provides real-time, high-contrast fluorescent outlining of the cystic duct and its junctions without the need for radiation, catheterization, or interruption of the surgical flow, factors that are commonly cited as sources of frustration with conventional X-ray cholangiography. Reduced background liver fluorescence further enhances visual clarity, which may increase surgeon confidence during critical dissection steps, particularly in anatomically challenging or inflamed cases. Collectively, these features contribute to improved perceived ease of use, smoother workflow integration, and greater overall surgeon satisfaction compared with more invasive or time-consuming imaging modalities. In the present study, intravenous ICG-FC achieved significantly higher surgeon satisfaction scores compared with the intracholecystic approach, underscoring its practicality, ease of use, and seamless integration into the operative workflow. Collectively, these features may enhance operative confidence and efficiency, particularly in cases with complex or distorted biliary anatomy. These findings may be particularly relevant in urgent or ambiguous clinical scenarios, where choledocholithiasis may coexist despite normal biochemical indicators, and rapid real-time anatomical clarification becomes crucial. where to

A major strength of this study lies in its prospective, randomized, three-arm design, which enabled a direct and standardized head-to-head comparison of conventional intraoperative cholangiography, intravenous indocyanine green fluorescence cholangiography, and intracholecystic indocyanine green fluorescence cholangiography under identical operative conditions. The relatively large and well-balanced sample size across groups, combined with strict application of the critical view of safety by experienced hepatobiliary surgeons, enhances the internal validity and reliability of the findings. Importantly, this study evaluated not only anatomical visualization outcomes but also operative efficiency, inflammatory response, and surgeon satisfaction, providing a comprehensive and clinically meaningful assessment of each imaging modality. The inclusion of surgeon-reported satisfaction using a standardized scale represents a particularly valuable contribution, as this aspect is infrequently addressed in the literature despite its relevance to real-world adoption and workflow integration. Collectively, these methodological strengths allow this study to offer robust comparative data and meaningful insights into the practical performance of fluorescence-based biliary imaging techniques during elective laparoscopic cholecystectomy.

However, several limitations that merit consideration should be acknowledged. First, although visualization rates were systematically recorded, assessment relied on intraoperative recognition of predefined anatomical landmarks rather than on objective quantitative fluorescence metrics, which may introduce a degree of subjectivity despite the use of standardized definitions. Second, surgeon satisfaction was evaluated immediately after surgery and may have been influenced by familiarity with fluorescence technology or personal preference, introducing potential performance or expectation bias that cannot be fully eliminated in procedural trials. An additional limitation concerns the assessment of surgeon satisfaction. Because blinding to the imaging modality was not feasible due to the inherent visual differences between techniques, surgeons were aware of the intervention used, which may have introduced subjective or expectation bias in satisfaction scoring. Although a standardized Likert scale was applied immediately postoperatively to minimize recall bias, the subjective nature of this endpoint should be considered when interpreting the superiority of fluorescence techniques in terms of surgeon satisfaction. Moreover, while all procedures were performed by experienced surgeons to reduce operator variability, this may limit generalizability to less experienced operators or training environments, where the relative benefits of each imaging modality could differ. Additionally, ICG was administered six hours prior to anesthesia at a dose of 0.3 mg/kg; it is possible that adjusting the timing or dosing could further improve visualization rates. Although a six-month follow-up was performed, it was based on clinical evaluation without systematic imaging or biochemical reassessment. Therefore, subclinical biliary abnormalities cannot be completely excluded, and longer-term structured follow-up studies would be required to definitively assess late biliary complications. In addition, the absence of predefined long-term endpoints should therefore be considered when interpreting the follow-up data.

Our cost analysis was limited to expendable supplies and did not account for equipment acquisition and maintenance. This study was restricted to elective procedures, limiting extrapolation to emergency settings. Finally, the single-center setting may reflect institutional expertise, equipment availability, and workflow characteristics that are not universally reproducible, underscoring the need for multicenter studies to validate these findings across diverse clinical settings and surgeon experience levels. Future research should focus on optimizing fluorescence protocols, expanding anatomical assessment, incorporating comprehensive cost analyses, and evaluating performance in acute and high-risk surgical scenarios.

## 5. Conclusions

Based on the presented findings, several clear conclusions can be drawn. Both intravenous and intracholecystic indocyanine green fluorescence cholangiography were non-inferior to conventional intraoperative X-ray cholangiography in visualizing key extrahepatic biliary structures, including the critical junction, cystic duct, common hepatic duct, and common bile duct, during elective laparoscopic cholecystectomy. Fluorescence-based techniques demonstrated superior procedural efficiency, with significantly shorter cholangiography times and higher surgeon satisfaction, reflecting improved usability and smoother integration into the operative workflow. Importantly, both fluorescence approaches were associated with high technical success rates and comparable perioperative safety profiles, without increasing postoperative complications or inflammatory burden.

Taken together, these results support the use of indocyanine green fluorescence cholangiography as a safe, effective, and user-friendly alternative to conventional X-ray cholangiography for intraoperative biliary mapping. Intravenous ICG appears particularly advantageous in routine elective cases due to its ease of application, while intracholecystic ICG may offer added value in selected scenarios requiring enhanced biliary contrast. Further multicenter studies and longer-term follow-up are warranted to refine indications, optimize protocols, and confirm generalizability across different surgical settings.

## Figures and Tables

**Table 1:** Baseline demographic and perioperative characteristics of patients undergoing elective laparoscopic cholecystectomy, stratified by cholangiography technique.

Variables		Group A (Standard IOC) *N*1 = 80	Group B (Intravenous ICG-FC) *N*2 = 80	Group C (Intracholecystic ICG-FC) *N*3 = 80	*p*-Value
Age (years), m ± sd		56.1 ± 12.9	49.9 ± 10.7	51.3 ± 10.3	0.359
Sex, No%	Female Male	57/80 (71.25%) 23/80 (28.75%)	48/80 (60.0%) 32/80 (24.0%)	50/80 (62.5%) 30/80 (37.5%)	0.642 -
BMI (kg/m^2^), m ± sd		28.3 ± 5.82	27.9 ± 3.51	29.5 ± 2.33	0.824
ASA Score, No %	II III	56/80 (70.0%) 24/80 (30.0%)	55/80 (68.75%) 25/80 (31.25%)	52/80 (65%) 28/80 (35%)	0.217 -
Indications, No%	Cholelithiasis Adenomyomatosis Polyps	76/80 (95%) 3/80 (3.75%) 1/80 (1.25%)	77/80 (96.25%) 1/80 (1.25%) 2/80 (2.50%)	77/80 (96.25%) 3/80 (3.75%) -	0.226 - -
Duration of surgical procedure (min), m ± sd		47.1 ± 7.31	46.5 ± 7.43	47.3 ± 6.86	0.858
Complications, No%	Yes No	4/80 (5.0%) 76/80 (95.0%)	3/80 (3.75%) 77/80 (96.25%)	2/80 (2.50%) 78/80 (97.5%)	0.573 -
Choledocholithiasis	Yes No	2/80 (2.50%) 78/80 (97.50%)	1/80 (1.25%) 79/80 (98.75%)	2/80 (2.50%) 78/80 (97.50%)	1.020 -

IOC: Standard intraoperative cholangiography; ICG-FC: Indocyanine green fluorescence cholangiography; m: mean; sd: standard deviation; No: number; ASA: American Society of Anesthesiologists.

**Table 2:** Visualization of extrahepatic biliary anatomy, cholangiography duration, and surgeon satisfaction according to the intraoperative biliary imaging technique.

Variables		Group A (Standard IOC) *N*1 = 80	Group B (Intravenous ICG-FC) *N*2 = 80	Group C (Intracholecystic ICG-FC) *N*3 = 80	*p*-Value
No. of failed investigations		3/80 (3.75%)	0/80 (0.0%)	0/80 (0.0%)	
Cholangiography time (s), m ± sd		305 ± 18.6	110 ± 11.1	125 ± 13.6	*p* < 0.001
Critical junction visualization	Yes No	71/77 (92.2%) 6/77 (7.8%)	73/80 (91.25%) 7/80 (8.75%)	76/80 (95%) 4/80 (5%)	0.737 -
CHD Visualization	Yes No	70/77 (90.9%) 7/77 (9.1%)	73/80 (91.25%) 7/80 (8.75%)	75/80 (93.75%) 5/80 (6.25%)	0.543 -
CBD Visualization	Yes No	71/77 (92.2%) 6/77 (7.8%)	77/80 (96.25%) 3/80 (3.75%)	79/80 (98.75%) 1/80 (1.25%)	0.106 -
CD Visualization	Yes No	72/77 (93.5%) 5/77 (6.5%)	78/80 (97.5%) 2/80 (2.5%)	77/80 (96.25%) 3/80 (3.75%)	0.234 -
Surgeon Satisfaction, m ± sd		2.9 ± 0.79	4.1 ± 1.05	4 ± 0.91	<0.001

IOC: Standard intraoperative cholangiography; ICG-FC: Indocyanine green fluorescence cholangiography; m: mean; sd: standard deviation; sec: seconds; No: number; CHD: common hepatic duct; CBD: common bile duct; CD: cystic duct.

**Table 3:** Preoperative and first postoperative day laboratory parameters, including hepatic, renal, coagulation, and inflammatory markers, stratified by intraoperative biliary imaging technique.

Variables	Group A (Standard IOC) *N1* = 80	Group B (Intravenous ICG-FC) *N2* = 80	Group C (Intracholecystic ICG-FC) *N3* = 80	*p*-Value
SGOT pre (g/dL), m ± sd	18.2 ± 3.82	20.7 ± 4.03	19.6 ± 4.07	0.472
SGOT post (g/dL), m ± sd	60.1 ± 15.5	60.1 ± 11.5	62.1 ± 10.3	1.016
SGPT pre (g/dL), m ± sd	18.2 ± 3.39	22.1 ± 3.41	21.9 ± 3.49	0.023
SGPT post (g/dL), m ± sd	66.2 ± 13.4	55.0 ± 15.2	58.5 ± 13.9	0.297
ALP pre (g/dL), m ± sd	87.2 ± 11.3	77.3 ± 11.4	80.7 ± 10.9	0.068
ALP post (g/dL), m ± sd	75.5 ± 12.3	65.0 ± 12.0	68.4 ± 10.4	0.073
γ- GT pre (g/dL), m ± sd	14.2 ± 2.97	14.9 ± 2.60	13.7 ± 3.01	0.637
γ- GT post (g/dL), m ± sd	10.0 ± 2.75	9.80 ± 2.04	10.3 ± 2.7	0.916
Indirect bilirubin pre (mg/dL), m ± sd	0.435 ± 0.248	0.365 ± 0.0880	0.37 ± 0.664	0.735
Indirect bilirubin post (mg/dL), m ± sd	0.556 ± 0.253	0.532 ± 0.0977	0.54 ± 0.024	0.727
Direct bilirubin pre (mg/dL), m ± sd	0.227 ± 0.110	0.211 ± 0.0314	0.225 ± 0.103	0.638
Direct bilirubin post (mg/dL), m ± sd	0.320 ± 0.132	0.154 ± 0.0381	0.129 ± 0.018	0.001
Total bilirubin pre (mg/dL), m ± sd	0.686 ± 0.367	0.576 ± 0.111	0.565 ± 0.241	0.377
Total bilirubin post (mg/dL), m ± sd	0.861 ± 0.287	0.686 ± 0.124	0.705 ± 0.224	0.093
PT pre (seconds), m ± sd	10.9 ± 0.584	11.1 ± 0.751	11.4 ± 0.789	0.724
PT post (seconds), m ± sd	11.1 ± 0.753	11.1 ± 0.643	11.2 ± −0.789	0.890
aPTT pre (seconds), m ± sd	27.4 ± 4.04	25.9 ± 2.21	26.2 ± 3.53	0.348
aPTT post (seconds), m ± sd	27.9 ± 3.82	26.8 ± 2.32	27.5 ± 2.9	0.682
INR pre (number), m ± sd	0.902 ± 0.0527	0.908 ± 0.0368	0.905 ± 0.0341	0.635
INR post (number), m ± sd	0.943 ± 0.0544	0.921 ± 0.404	0.955 ± 0.3502	0.428
Urea pre (mg/dL), m ± sd	32.2 ± 9.31	30.6 ± 6.43	31.4 ± 4.42	0.470
Urea post (mg/dL), m ± sd	27.3 ± 9.31	34.1 ± 10.5	35 ± 5.8	0.443
Creatinine pre (mg/dL), m ± sd	0.713 ± 0.117	0.843 ± 0.145	0.815 ± −0.125	0.040
Creatinine post (mg/dL), m ± sd	0.758 ± 0.186	0.726 ± 0.179	0.755 ± 0.201	0.780
WBC pre (mm^3^/L), m ± sd	5.92 ± 1.74	5.08 ± 0.942	5.86 ± 0.866	0.274
WBC post (mm^3^/L), m ± sd	9.02 ± 2.20	7.33 ± 1.13	7.89 ± 1.76	0.045
CRP pre (mg/dL), m ± sd	0.610 ± 0.292	0.670 ± 0.250	0.738 ± 0.341	0.858
CRP post (mg/dL), m ± sd	3.20 ± 2.42	5.05 ± 1.83	4.08 ± 1.57	0.174
TNF-a pre (pg/dL), m ± sd	1.99 ± 0.223	2.06 ± 0.276	3.05 ± 0.352	0.567
TNF-a post (pg/dL), m ± sd	2.09 ± 0.195	2.16 ± 0.227	2.15 ± 0.335	0.365
IL-6 pre (pg/dL), m ± sd	0.910 ± 0.0745	0.895 ± 0.0642	0.84 ± 0.036	0.235
IL-6 post (pg/dL), m ± sd	0.949 ± 0.0863	0.924 ± 0.0648	0.946 ± 0.288	0.535

## Data Availability

The original contributions presented in this study are included in this article. Further inquiries can be directed to the corresponding author(s).

## Abbreviations

The following abbreviations are used in this manuscript:

ASA	American Society of Anesthesiologists
aPTT	Activated Partial Thromboplastin Time
BDI	Bile Duct Injury
BMI	Body Mass Index
CBD	Common Bile Duct
CD	Cystic Duct
CHD	Common Hepatic Duct
CRP	C-Reactive Protein
CT	Computed Tomography
CVS	Critical View of Safety
ERAS	Enhanced Recovery After Surgery
ERCP	Endoscopic Retrograde Cholangiopancreatography
ICG	Indocyanine Green
ICG-FC	Indocyanine Green Fluorescence Cholangiography
IOC	Intraoperative Cholangiography
IL-6	Interleukin-6
INR	International Normalized Ratio
LC	Laparoscopic Cholecystectomy
MRCP	Magnetic Resonance Cholangiopancreatography
NIR	Near-Infrared
NIR-ICG	Near-Infrared Indocyanine Green
NIRC	Near-Infrared Cholangiography
PT	Prothrombin Time
RCT	Randomized Controlled Trial
TNF-α	Tumor Necrosis Factor-alpha
WBC	White Blood Cell (count)
